# Neural correlates of moral and non-moral emotion in female psychopathy

**DOI:** 10.3389/fnhum.2014.00741

**Published:** 2014-09-25

**Authors:** Carla L. Harenski, Bethany G. Edwards, Keith A. Harenski, Kent A. Kiehl

**Affiliations:** ^1^The MIND Research NetworkAlbuquerque, NM, USA; ^2^Departments of Psychology and Neuroscience, University of New MexicoAlbuquerque, NM, USA

**Keywords:** emotion, moral, fMRI, psychopathy, female, amygdala, anterior cingulate

## Abstract

This study presents the first neuroimaging investigation of female psychopathy in an incarcerated population. Prior studies have found that male psychopathy is associated with reduced limbic and paralimbic activation when processing emotional stimuli and making moral judgments. The goal of this study was to investigate whether these findings extend to female psychopathy. During fMRI scanning, 157 incarcerated and 46 non-incarcerated female participants viewed unpleasant pictures, half which depicted moral transgressions, and neutral pictures. Participants rated each picture on moral transgression severity. Psychopathy was assessed using the Psychopathy Checklist-Revised (PCL-R) in all incarcerated participants. Non-incarcerated participants were included as a control group to derive brain regions of interest associated with viewing unpleasant vs. neutral pictures (emotion contrast), and unpleasant pictures depicting moral transgressions vs. unpleasant pictures without moral transgressions (moral contrast). Regression analyses in the incarcerated group examined the association between PCL-R scores and brain activation in the emotion and moral contrasts. Results of the emotion contrast revealed a negative correlation between PCL-R scores and activation in the right amygdala and rostral anterior cingulate. Results of the moral contrast revealed a negative correlation between PCL-R scores and activation in the right temporo-parietal junction. These results indicate that female psychopathy, like male psychopathy, is characterized by reduced limbic activation during emotion processing. In contrast, reduced temporo-parietal activation to moral transgressions has been less observed in male psychopathy. These results extend prior findings in male psychopathy to female psychopathy, and reveal aberrant neural responses to morally-salient stimuli that may be unique to female psychopathy.

## Introduction

Psychopathy is a serious mental health disorder defined by a cluster of interpersonal, affective, and behavioral characteristics including impulsivity, grandiosity, and callousness (Hare, 2003). A core feature of psychopathy is an inability to experience a normal range and depth of emotions, including “moral emotions” such as guilt and empathy. These characteristics are early-emerging, severe, and persist into adulthood (Lynam et al., 2007).

Deficits in emotion processing related to psychopathy have been demonstrated across multiple modalities. Psychopathic males show reduced physiological responses to unpleasant and fear-inducing events (Patrick et al., 1993, 1994), impaired ability to identify multiple types of facial and vocal expressions (Blair et al., 2002; Kosson et al., 2002; Glass and Newman, 2006; Bagley et al., 2009; Dawel et al., 2012), and reduced response facilitation to emotional words in lexical decision tasks (Lorenz and Newman, 2002). Fewer investigations of these processes have been conducted in female psychopathy. One study found that low-anxious psychopathic females showed reduced startle potentiation to unpleasant images (Sutton et al., 2002). Another study found that psychopathic and non-psychopathic females performed similarly on tasks evaluating response facilitation to emotional words in a lexical decision task, and response inhibition in a passive avoidance learning task (Vitale et al., 2011). These results suggest that female and male psychopathy share some, but not all, of the same aberrant emotional responses.

Neuroimaging studies have provided additional support of emotional dysfunction in psychopathy. Psychopathic males show reduced engagement of brain regions associated with emotion processing including the amygdala, anterior cingulate/ACC, and ventromedial prefrontal cortex/vmPFC when viewing emotional words, conditioned fear stimuli, statements, or pictures depicting immoral behavior, and during emotional perspective taking tasks (Kiehl et al., 2001; Birbaumer et al., 2005; Glenn et al., 2009; Harenski et al., 2010a; Decety et al., 2013). Individuals with high levels of callous-unemotional traits show reduced amygdala activation to fearful facial expressions (Marsh and Blair, 2008; Jones et al., 2009), and psychopathic males also show reduced vmPFC and orbitofrontal cortex activation to several types of emotional facial expressions (Decety et al., 2014). Regarding females, neuroimaging studies have reported negative correlations between self-reported psychopathic traits and amygdala responses to fearful facial expressions (Carre et al., 2013) and unpleasant pictures (Harenski et al., 2009). These results suggest that female psychopathy, like male psychopathy, may be associated with reduced engagement of limbic regions during affective tasks. However, this hypothesis has not been tested in females with clinical levels of psychopathy.

Emotion deficits and associated brain dysfunction in psychopathy are believed to impair moral development. Blair (2007) proposed a neurodevelopmental account of psychopathy in which early dysfunction within the amygdala and vmPFC contributes to impaired moral socialization. The proposal is based on the importance of stimulus-reinforcement associations in moral socialization (learning that certain behaviors are harmful to others and should be avoided) and the role of the amygdala (valence, i.e., “good”/“bad” representation) and vmPFC (outcome expectancy) in these processes. Consistent with this hypothesis, psychopathic males have shown reduced vmPFC and amygdala engagement when presented with pictures depicting immoral behaviors and complex moral dilemmas (Glenn et al., 2009; Harenski et al., 2010a).

Whether the neuroimaging findings in male psychopathy summarized above extend to female psychopathy is unknown. We used fMRI to scan a large sample of incarcerated females with varying levels of psychopathy, and a comparison group of non-incarcerated females, while they viewed unpleasant pictures that did or did not depict moral transgressions and thematically-matched neutral pictures. They also rated the severity of moral transgressions. We predicted that psychopathy would be negatively correlated with brain activation while viewing unpleasant relative to neutral pictures in the ACC, amygdala, and vmPFC. We further predicted that activation within the latter two regions would be negatively correlated with psychopathy when viewing unpleasant pictures depicting moral transgressions relative to pictures without moral transgressions.

Regarding the moral salience aspect of the picture task used in this study, the task has been shown to reliably engage brain regions frequently implicated in moral judgment in non-clinical populations (Harenski and Hamann, 2006; Harenski et al., 2008, 2012). Such regions include the amygdala and vmPFC, and additionally the temporo-parietal junction [which supports several component cognitive processes related to inferring mental states (Decety and Lamm, 2007)], posterior cingulate and precuneus (Young and Dungan, 2012). Whether engagement of these latter regions (i.e., beyond vmPFC and amygdala) would show psychopathy-related differences to moral stimuli was an open question.

## Materials and methods

### Participants

The study included 164 incarcerated female volunteers recruited from a medium-security correctional facility, and 46 non-incarcerated female volunteers, who met inclusion criteria. Inclusion criteria were: age between 18 and 50, reading level above 4th grade, IQ above 75, no history of seizures, no current Diagnostic and Statistical Manual of Mental Disorders (4th ed.) Axis I diagnosis, and no lifetime history of a psychotic disorder in self or first-degree relative. Non-incarcerated participants were excluded if they had any history of alcohol or drug disorder.

Seven incarcerated participants were excluded from analysis due to poor task performance (e.g., drowsiness observed with real-time eyetracking, *n* = 3), or equipment malfunction/experimenter error (*n* = 4). The final sample included 157 incarcerated and 46 non-incarcerated participants. Demographic characteristics of each group are provided in Table 1.

**Table 1:** **Descriptive statistics and group differences between non-incarcerated and incarcerated participants**.

	**Non-incarcerated (*n* = 46)**			**Incarcerated (*n* = 157)**			***t***	***p***
	***M***	***SD***	**Range**	***M***	***SD***	**Range**		
Age	27.0	10.58	18–50	33.2	6.47	21–49	4.89	<0.001
IQ	115.7	10.42	94–137	96.4	10.58	77–131	11.19	<0.001
SDD^a^	0	0	–	1.9	1.29	0–6	10.53	<0.001
Moral rating	4.1	0.59	1.9–5.0	3.9	0.54	2.8–4.7	2.02	0.045
Non-moral rating	2.6	0.75	1.1–4.5	2.1	0.55	1.2–3.8	4.22	<0.001
Neutral rating	1.5	0.27	1.0–2.6	1.5	0.31	1.1–3.0	0.94	0.35
PCL-R total	–	–	–	18.7	6.06	3.2–32.6	–	–
Factor 1	–	–	–	4.3	2.64	0–11	–	–
Factor 2	–	–	–	12.2	3.62	2.2–20	–	–

a *SDD, Total number of lifetime substance dependence diagnoses*.

All participants completed the research version of the Structured Clinical Interview for DSM Disorders (SCID) (First et al., 2002) to assess past and present Axis I and II disorders. This included the substance use disorder screening questionnaire and module, which were used to evaluate alcohol and drug disorder histories for exclusion purposes (non-incarcerated participants) and quantify prior substance use severity for data analysis (incarcerated participants). The latter was accomplished by counting the total number of lifetime alcohol and drug dependence diagnoses. Substance use disorders accounted for the majority of past Axis I disorders across incarcerated participants, followed by major depression. See Table S1 in the online data supplement for a complete summary of Axis I and II disorders across participants. IQ was evaluated using the Wechsler Adult Intelligence Scale (Wechsler, 1997).

Psychopathy was assessed in all incarcerated participants using the Hare Psychopathy Checklist-Revised (PCL-R) (Hare, 2003). The PCL-R is a reliable and valid instrument for the assessment of psychopathy (Hart and Hare, 1989) involving a semi-structured interview covering school, employment, relationship and criminal history, and a review of the participant's institutional records. The PCL-R is scored on 20 items measuring personality and behavior characteristics, each with a 3-point severity scale (0, 1, or 2). Total scores range from 0 to 40. Fifteen percent of all PCL-R assessments were independently scored by a second rater (ICC = 0.93). These assessments were similar in score distribution of the entire incarcerated group (*M* = 19.3, *SD* = 6.86, Range = 5–31). PCL-R assessments were not conducted in non-incarcerated participants.

Written informed consent was obtained from all participants, after a complete description of the study procedures. The study was reviewed and approved by the University of New Mexico Human Research Review Committee. Participants received monetary compensation for participation. Incarcerated participants were paid at a rate commensurate to work assignments at their facility.

### Task

Three picture sets (25 moral, 25 non-moral, 25 neutral) were created using the International Affective Picture System (Lang et al., 1995) and media sources. Moral pictures depicted unpleasant scenes indicating a moral transgression (e.g., a drunk driver). Non-moral pictures depicted unpleasant scenes without moral content (e.g., an angry driver). Neutral pictures depicted scenes without moral content (e.g., a normal driver). Moral and non-moral pictures were matched on emotional arousal and social complexity, and were matched to neutral pictures on social complexity (the matching procedure is described in detail elsewhere) (Harenski et al., 2008, 2010b). Matching on social content helped ensure that there were similar numbers of faces and bodies in the different conditions, which have been shown to differentially engage brain regions such as the temporo-parietal junction (Kret et al., 2011).

Participants rated the severity of moral transgression in each picture from 1 (no transgression) to 5 (high transgression severity). Pictures were displayed for six seconds, followed by a four-second rating scale in which a moving red bar progressed from 1 to 5. The participant pressed a button to stop the bar when it reached their desired rating. This format was chosen for simplicity (pressing one rather than several buttons). Following each rating, a four-second delay preceded the next trial. Moral, non-moral, and neutral trials were randomized along with 25 “jitter” fixation trials (10 seconds) randomly interspersed between picture trials. The 100 trials (25 moral, 25 non-moral, 25 neutral, 25 fixation) were presented across two runs.

### Image acquisition and analysis

MR images were collected using a mobile Siemens 1.5T Avanto with advanced SQ gradients (max slew rate 200T/m/s, 346T/m/s vector summation, rise time 200 us) equipped with a 12-element head coil. The EPI gradient-echo pulse sequence (TR/TE 2000/39 ms, flip angle 90°, FOV 24 × 24 cm, 64 × 64 matrix, 3.4 × 3.4 mm in-plane resolution, 5 mm slice thickness, 30 slices) effectively covered the entire brain (150 mm) in 2.0 s. Head motion was minimized using padding and restraint.

To correct residual head motion, “bad” images (confounded by motion or radio-frequency spikes) were estimated and removed using ART-Repair (Mazaika et al., 2007). These images were determined by calculating the mean intensity for a given time series and identifying individual images whose intensity was greater than four standard deviations from the mean. The offending image(s) were replaced in the time series by a rolling mean image, and regressed in the statistical model. The mean number of images removed across participants was 5.5 (of 712).

Imaging data were analyzed using SPM5 (www.fil.ion.ucl.ac.uk/spm/software/spm5). Functional images were spatially normalized to the MNI template and smoothed (8 mm FWHM). Picture presentations (moral, non-moral, neutral) and the rating period for all pictures were modeled as four separate events. Each event was modeled with a six (picture) or four (rating) second hemodynamic response function. Functional images were computed for each participant that represented brain activation associated with viewing moral, non-moral, or neutral pictures. The moral + non-moral > neutral comparison evaluated brain activation to emotional pictures regardless of moral content. The moral > non-moral picture comparison evaluated brain activation to morally-salient pictures while controlling for emotional content. Participants' severity of moral transgression ratings of each picture were included as covariates of no interest to model variance associated with individual differences in ratings. PCL-R scores were entered into a regression with individual moral + non-moral > neutral contrast images, and a separate regression with individual moral > non-moral contrast images. PCL-R Factor 1 and Factor 2 scores were also entered into two separate regression analyses, one for each contrast (Factor 1 and Factor 2 scores were positively correlated at *r* = 0.47).

Age and IQ were not significantly correlated with PCL-R or Factor scores. PCL-R and Factor 2 scores were significantly correlated with number of substance dependencies (*r* = 0.25, 0.31, respectively). Substance use severity is associated with reduced functioning in brain regions that overlap with psychopathy (Childress et al., 1999; Thompson et al., 2004). However, substance use may also be considered an integral aspect of psychopathy, thus it would not necessarily be informative to remove substance use-related effects from the results. To thoroughly examine psychopathy and substance-use related effects, we present all analyses with and without substance dependence included as a covariate.

Analyses were performed on a voxel-by-voxel basis over the entire brain using the general linear model in SPM5. Thresholds for whole-brain family-wise error multiple comparison correction were determined using AlphaSim (Ward, 2000). Hypotheses were also tested in regions of interest (amygdala, vmPFC, ACC). Peak coordinates for these regions were drawn from the moral + non-moral > neutral and moral > non-moral functional maps of the 46 non-incarcerated participants. In all a priori regions of interest, family-wise error extent thresholds were small-volume corrected using 10 mm spheres surrounding the peak coordinate.

## Results

### Behavioral results

All participants rated moral pictures (*M* = 4.0, *SD* = 0.59) higher on transgression severity than non-moral [*M* = 2.4, *SD* = 0.74; *F*_(203)_ = 1109.8, *p* < 0.0001] and neutral [*M* = 1.5, *SD* = 0.29; *F*_(203)_ = 3638.7, *p* < 0.0001] pictures. Non-moral pictures were also rated higher on transgression severity than neutral pictures [*F*_(203)_ = 478.8, *p* < 0.0001], consistent with our prior studies (Harenski et al., 2008, 2010a) and likely due to the unpleasant content of non-moral relative to neutral pictures. Non-incarcerated participants rated moral and non-moral, but not neutral, pictures higher on transgression severity compared to incarcerated participants (Table 1). Moral, non-moral, and neutral picture ratings in the incarcerated sample were not significantly correlated with PCL-R scores, Factor 1 scores, or Factor 2 scores (all *r*'s < 0.09).

### Imaging results

**Figure 1:**
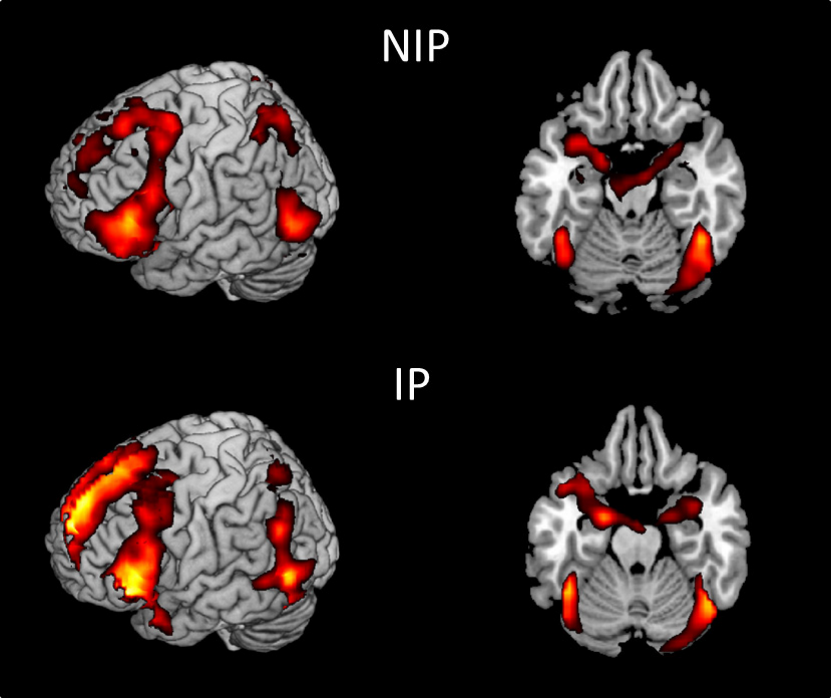
**Main effect of viewing moral + non-moral vs. neutral pictures in non-incarcerated (NIP; *n* = 46), and incarcerated (IP; *n* = 157) participants (*p* < 0.001, uncorrected)**.

**Figure 2:**
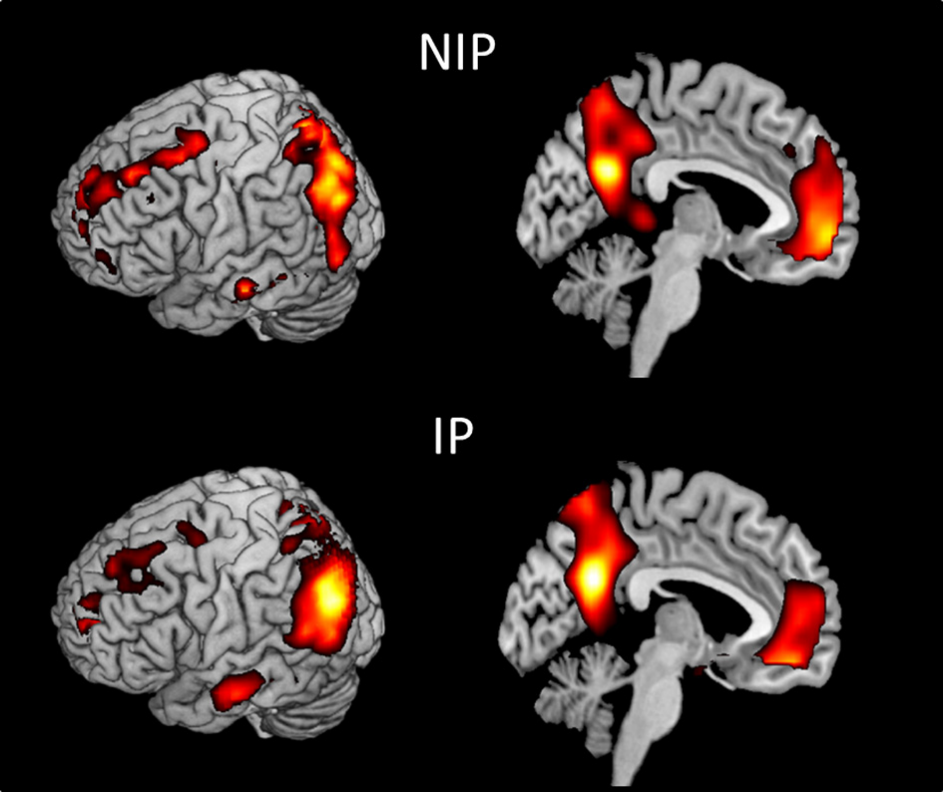
**Main effect of viewing moral vs. non-moral pictures in non-incarcerated (NIP; *n* = 46), and incarcerated (IP; *n* = 157) participants (*p* < 0.001, uncorrected)**.

We first examined the main effects of viewing moral and non-moral > neutral (Figure 1) and moral > non-moral (Figure 2) pictures across all non-incarcerated and incarcerated participants. The main effect of moral + non-moral vs. non-moral pictures revealed increased hemodynamic responses in limbic and paralimbic regions including the amygdala, medial prefrontal cortex, and anterior cingulate, as well as ventrolateral prefrontal cortex. The main effect of moral vs. non-moral pictures revealed increased hemodynamic responses in regions previously implicated in moral judgment (Greene and Haidt, 2002; Moll et al., 2005; Raine and Yang, 2006) including the ventromedial prefrontal cortex, temporo-parietal junction, and posterior cingulate. Results were largely similar across incarcerated and non-incarcerated groups.

**Figure 3:**
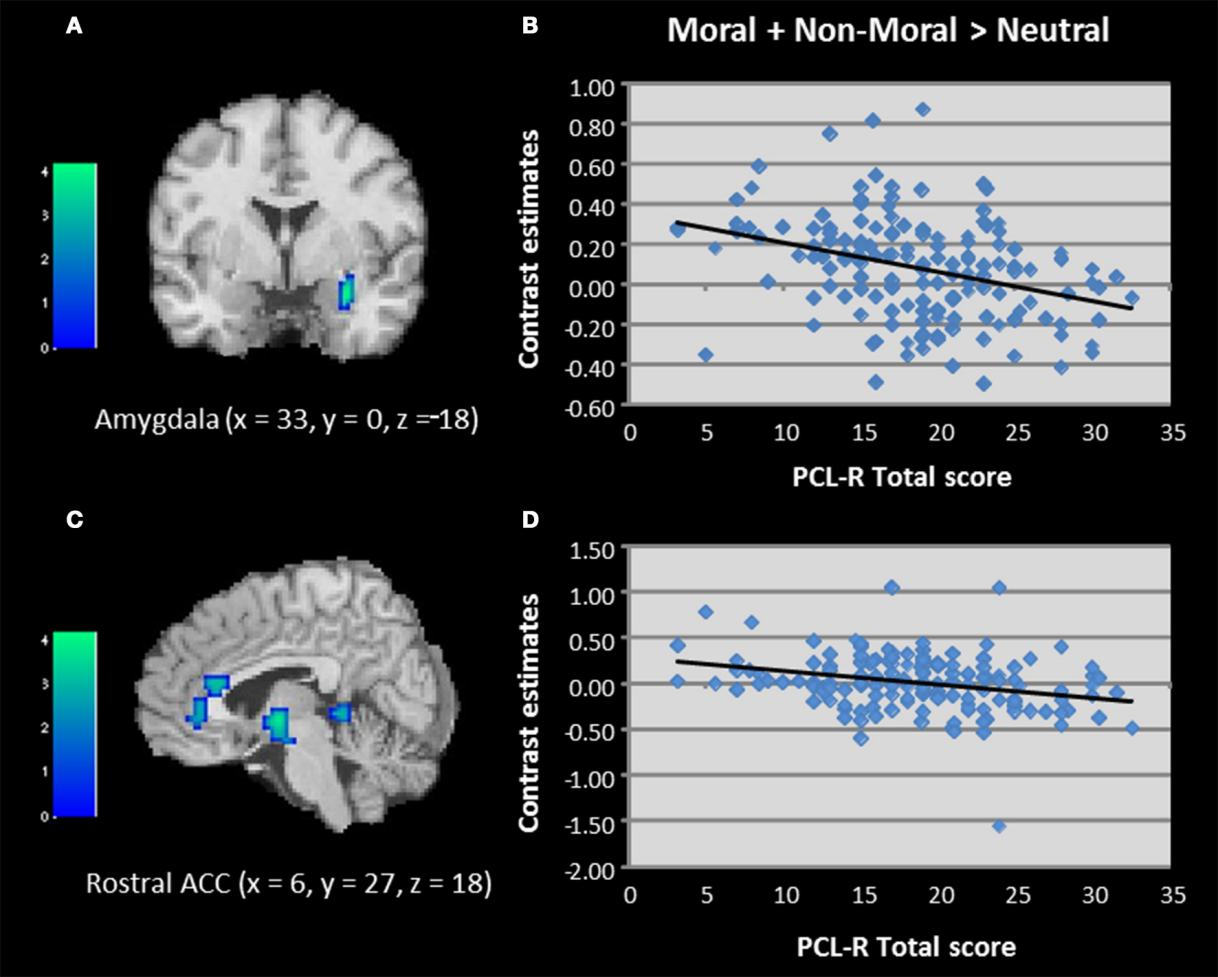
**(A–D)** Correlations between total PCL-R scores and activation in the amygdala and rostral anterior cingulate when viewing unpleasant relative to neutral pictures in incarcerated individuals (*N* = 157). Contrasts estimates represent differential magnitude of associations between hemodynamic response and the statistical model during moral vs. non-moral picture presentations. Color bars represent *t*-values. Image thresholded at *p* < 0.001, uncorrected.

Regression analyses were used to examine the relationship between PCL-R scores and brain activation to moral + non-moral vs. neutral pictures across all incarcerated participants. This revealed a negative correlation between PCL-R scores and activation in the right amygdala and rostral ACC (Table 2; Figure 3). Negative correlations were also present in superior and inferior temporal cortex. The regression analysis with Factor 1 and Factor 2 scores showed that the negative correlation with right amygdala and rostral ACC activity was related to Factor 2 but not Factor 1 scores (Table 2).

**Table 2:** **Correlations between hemodynamic responses and PCL-R scores among incarcerated participants (*n* = 157) in the moral + non-moral vs. neutral and moral > non-moral picture comparison^a^**.

	***BA***	***x***	***y***	***z***	***t*-value**	***k***	***p*^b^**
**MORAL + NON-MORAL > NEUTRAL**							
**PCL-R positive**							
No correlations							
**PCL-R negative**							
R. amygdala		33	0	−18	4.23	22	0.003^c^
R. rostral anterior cingulate	24	6	27	18	3.48	63	0.03
*L. superior temporal gyrus	41	−57	−45	9	3.81	68	–
R. fusiform gyrus	37	42	−57	−24	3.90	30	–
L. superior temporal gyrus	41	−57	−45	9	3.81	68	–
**Factor 1**							
No positive or negative correlations							
**Factor 2 positive**							
No correlations							
**Factor 2 negative**							
*R. amygdala		33	0	−15	4.04	28	0.005
R. rostral anterior cingulate	24	6	24	21	3.66	104	0.016
		3	39	6	4.41	50	–
R. temporo-parietal junction	39	51	−63	33	4.34	75	–
L. posterior cingulate	29	−9	−42	6	4.04	32	–
L. precuneus	7	−3	−66	57	3.63	40	–
**MORAL > NON-MORAL**							
**PCL-R positive**							
No correlations							
**PCL-R negative**							
R. temporo-parietal junction	22/39	60	−54	15	4.39	52	–
*R. inferior occipital gyrus	18	30	−90	−12	4.26	57	–
L. fusiform gyrus	19	−39	−69	−12	4.13	29	–
L. parahippocampal gyrus	36	−33	−30	−21	3.91	22	–
R. postcentral gyrus	40	60	−30	18	3.76	45	–
**Factor 1 positive**							
No correlations							
**Factor 1 negative**							
L. parahippocampal gyrus	19	−21	−54	−12	4.07	39	–
**Factor 2**							
No positive or negative correlations							

a *Asterisks denote results which were no longer significant after substance dependence was included as a covariate*.

*BA, Brodmann Area; k, cluster size (voxels). t-values reported for all effects using beta values from GLM regression analyses. Coordinates refer to Montreal Neurological Institute space*.

b *Small-volume corrected p-values listed for regions of interest. Other regions are significant at p < 0.05, corrected*.

c *The correlation in the amygdala was also significant at the whole-brain corrected threshold*.

**Figure 4:**
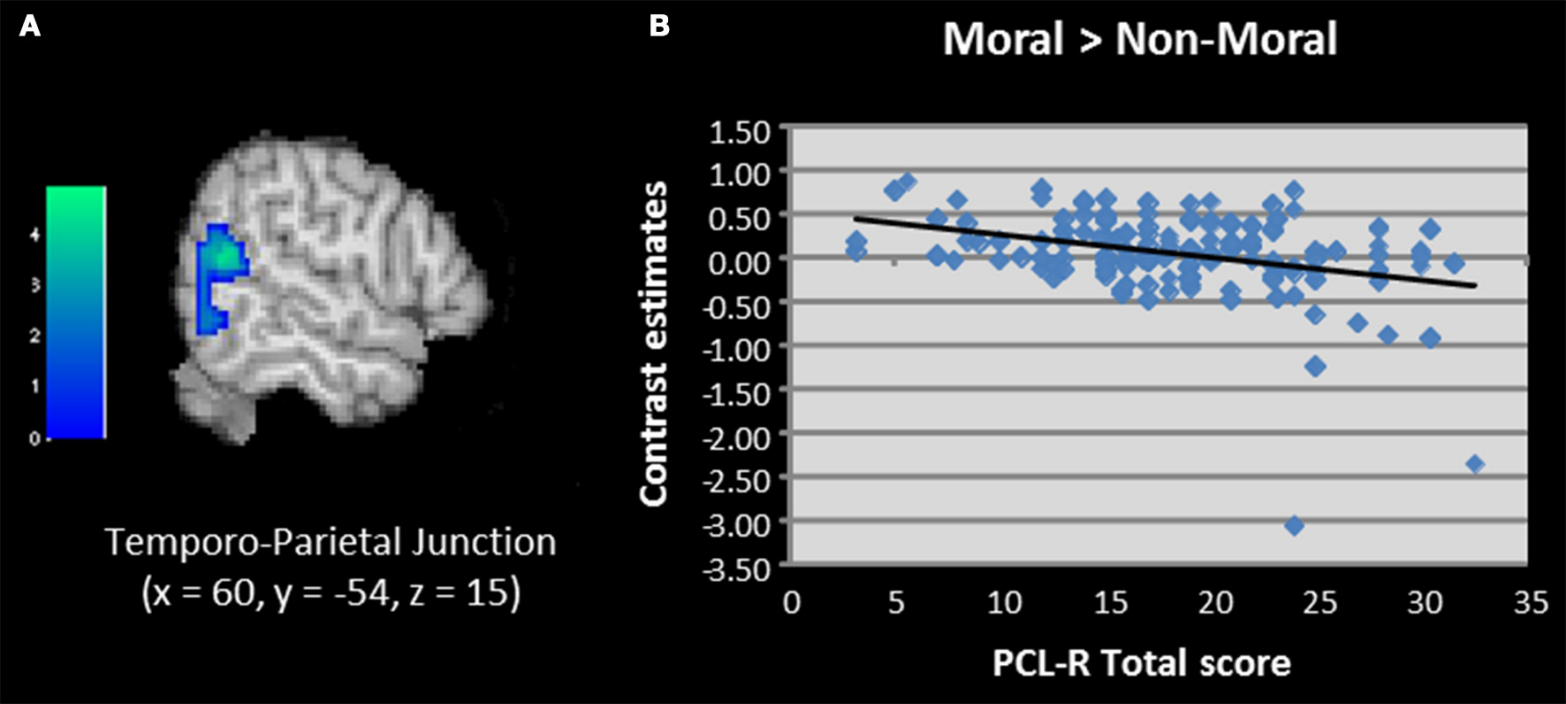
**(A,B)** Correlation between total PCL-R scores and activation in the temporo-parietal junction when viewing unpleasant moral relative to unpleasant non-moral pictures in incarcerated individuals (*N* = 157). Contrasts estimates represent differential magnitude of associations between hemodynamic response and the statistical model during moral vs. non-moral picture presentations. Color bars represent *t*-values. Image thresholded at *p* < 0.001, uncorrected.

We next used regression to examine correlations between PCL-R scores and brain activation to moral vs. non-moral pictures. This analysis revealed a negative correlation between PCL-R scores and activation in the right temporo-parietal junction/TPJ (Table 2; Figure 4). Additional negative correlations were present in the parahippocampal and fusiform gyrus. Factor scores were not significantly correlated with any regions of interest; however, the negative correlation between total PCL-R scores and parahippocampal gyrus activation was related to Factor 1 but not Factor 2 scores.

All of the above results remained significant when substance dependence was included as a covariate, with the exception of the correlation between Factor 2 scores and right amygdala.

## Discussion

This study examined the neurobiology of emotion and moral judgment in female psychopathy. In accordance with hypotheses and prior studies of male psychopathy, PCL-R scores were negatively correlated with activation to unpleasant pictures in the right amygdala and rostral ACC. PCL-R scores were also negatively correlated with activation to unpleasant pictures depicting moral transgressions in the right TPJ. These results extend prior findings regarding emotion processing in adult male psychopathy to female psychopathy, and reveal aberrant neural responses to morally-salient stimuli that may be unique to female psychopathy.

Reduced amygdala engagement during emotional processing is a consistent finding in male psychopathy, in such tasks as viewing emotional words, conditioned fear, making moral judgments, and during affective perspective taking (Kiehl et al., 2001; Birbaumer et al., 2005; Glenn et al., 2009; Harenski et al., 2010a; Decety et al., 2013). Anatomical imaging studies of male psychopathy have also reported reduced amygdala gray matter volume (Yang et al., 2009; Ermer et al., 2012). While the anatomical findings implicated bilateral amygdala, the present findings and those of prior fMRI studies have often been lateralized to the right (Harenski et al., 2010a; Decety et al., 2013) or left (Birbaumer et al., 2005; Glenn et al., 2009) amygdala, though one study reported bilateral effects (Kiehl et al., 2001). Because this is the first fMRI study of female psychopathy in an incarcerated population, it would be premature to conclude that reduced amygdala activation to emotional stimuli is right-lateralized, especially given the focus of this study on moral judgment. Whether these findings extend to other emotion paradigms is a question for future studies.

The analysis of PCL-R factor scores revealed a negative correlation between right amygdala activation to unpleasant pictures and Factor 2 scores. This is in contrast to prior male psychopathy studies in which negative associations with amygdala activation were primarily related to Factor 1 scores (Harenski et al., 2010a; Decety et al., 2013). However, the correlation was no longer significant when substance dependence was included as a covariate. This is likely due to predictor criterion overlap (i.e., substance abuse is a symptom of psychopathy and measured most directly by PCL-R Factor 2 items). Additionally, the negative correlation between right amygdala activation and total PCL-R scores was significant regardless of the inclusion of substance dependence as a covariate.

Relative to the amygdala, the status of ACC function in psychopathy is less clear. ACC dysfunction is proposed in some theoretical models of psychopathy (Kiehl, 2006) but not others (Blair, 2005). Anatomical and functional imaging studies of the ACC in male psychopathy have produced mixed findings. Anatomical studies have not reported reductions in ACC volume (Glenn et al., 2010; Ermer et al., 2012), whereas functional imaging studies have found reduced rostral ACC responses during emotion tasks (Kiehl et al., 2001; Birbaumer et al., 2005). Here we observed a negative correlation between PCL-R scores and rostral ACC activation to unpleasant pictures. The region of rostral ACC that was correlated with psychopathy corresponds to the rostral section of the anterior mid cingulate cortex/aMCC, bordering the pregenual anterior cingulate. The aMCC has been implicated in studies examining a wide variety of negative emotion and cognitive control processes and has extensive connections with subcortical brain regions including the amygdala and ventral striatum (Shackman et al., 2011). One possible explanation for functional but not anatomical psychopathy-related differences in this region is that it may be anatomically intact in psychopathy but less responsive when processing emotional stimuli due to reduced input from one or more of the emotional processing brain regions with which it is connected (e.g., amygdala).

Similar to the findings in the right amygdala, the analysis of PCL-R factor scores revealed a negative correlation between rostral ACC activation to unpleasant pictures and Factor 2 scores. Unlike the findings in the amygdala, this result was unaffected by substance dependence. Prior psychopathy studies that reported reduced ACC function did not examine associations with Factor scores (e.g., Kiehl et al., 2001; Birbaumer et al., 2005). Thus, it is unknown whether the Factor 2-ACC negative correlation during emotion processing is unique to female psychopathy or also present in male psychopathy. More research in both male and female psychopathy is needed to explore this possibility. Overall, the current results suggest that reduced ACC function during emotion processing is related primarily to the lifestyle/antisocial aspects of female psychopathy. It is also worth noting that PCL-R Factor 2 contains items relevant to the developmental nature of psychopathy, including childhood, adolescent, and adult-related disruptive behavior; thus the effects related to Factor 2 may be developmental in nature.

The comparison of moral vs. non-moral pictures yielded results that were less consistent with prior findings in male psychopathy. PCL-R scores were negatively correlated with right TPJ activation. In our prior study of male psychopathy using the same moral judgment task, psychopathy was not associated with reduced (or increased) TPJ responses when viewing moral pictures (Harenski et al., 2010a). However, psychopathic males showed a positive correlation between right TPJ activation and severity of moral transgression ratings. Non-psychopathic males, in contrast, showed no correlation between right TPJ and severity ratings. Drawing from neuroimaging and lesion evidence regarding the role of the right TPJ in moral judgment (Greene and Haidt, 2002; Moll et al., 2005; Raine and Yang, 2006), particularly in attributing intentions to moral transgressions (Young et al., 2010; Koster-Hale et al., 2013), we proposed that intention attributions influenced severity of moral transgression ratings more in psychopathic males relative to non-psychopathic males. In the present study, female psychopathy was associated with less engagement of the right TPJ when viewing all moral pictures (regardless of how moral transgressions were rated) which could indicate a generally reduced sensitivity to the intentionality aspect of moral transgressions.

Although the role of the TPJ in making intentionality attributions during moral judgment has been well-demonstrated, studies have highlighted other functions of this region. For example, the right TPJ has been implicated in sense of agency, self-other discrimination, and directing attention to task-relevant stimuli (Decety and Lamm, 2007; Mitchell, 2008). Decety and Lamm (2007) proposed that rTPJ is associated with a variety of lower-level processes (e.g., redirection of attention) that contribute to higher level functions such as mentalizing. Thus, lower TPJ activation in psychopathic females could be related to intention attribution and/or other TPJ-related processes. The moral pictures contained multiple cues that could be differentially attended to by high and low psychopathy females. In other words, specific components of the pictures that are considered relevant to moral judgment, and the amount of attention paid to those components, may differ as a function of psychopathy. Future studies employing methods such as eye tracking could shed light on this possibility. Right TPJ activation has also shown elevated activation in individuals with heightened justice sensitivity as well as in individuals more likely to exculpate harmful actions resulting from innocent intentions (Young and Saxe, 2009; Yoder and Decety, 2014). It is possible that females higher in psychopathy have reduced justice sensitivity, which may be emphasized when evaluating moral transgressions and related to TPJ activation. This possibility requires further study. Regarding the forgiveness of unintentional moral transgressions, psychopathic males are in fact more likely than non-psychopathic males to exhibit this behavior (Young et al., 2012). However, whether psychopathic females are similar to psychopathic males in this respect has not been investigated.

PCL-R scores were unrelated to behavioral moral judgments. This result is in line with prior findings in male psychopathy using the same task (Harenski et al., 2010a), and other studies showing similar moral judgments in psychopathic and non-psychopathic males (Cima et al., 2010; Aharoni et al., 2012). However, male psychopathy-related differences in certain types of moral judgment have been reported (Koenigs et al., 2012; Young et al., 2012). The moral task used in this study was relatively straightforward compared to other tasks such as moral dilemmas. Female psychopathy may be associated with abnormalities in the latter type of moral judgment.

Limitations of this study should be considered. The standard PCL-R cutoff score for psychopathy is 30/40. Eight participants in this study scored 30 or higher on the PCL-R. We might have observed stronger and/or additional correlations between brain activation and PCL-R scores if more 30+ participants were included. Previous studies used lower PCL-R cutoff scores (e.g., 24) to identify female psychopathy (Vitale et al., 2007, 2011) due to lower base rates of psychopathy relative to males at the 30 cutoff score (Vitale et al., 2002) and lower levels of criminal behavior in females than males (Goldstein et al., 1996). Regarding our emotion task, it should be emphasized that participants made moral judgments of all pictures. We previously demonstrated that judging morally-salient pictures on severity of moral transgression, relative to making indoor/outdoor judgments of the same pictures, was associated with greater activation in the vmPFC, a region prominent in neurodevelopmental theories of psychopathy (Harenski et al., 2010b). It is possible that using a more “implicit” emotional task not related to moral judgment could yield additional/different findings.

In conclusion, we demonstrated that females with clinical levels of psychopathy show reduced amygdala and ACC activation when processing emotional stimuli, similar to findings in male psychopathy. Female psychopathy was also associated with aberrant right TPJ activation related to moral judgments, but the results differed from prior findings in male psychopathy. Overall, the results provide new evidence of neurobiological dysfunction in female psychopathy and support theories of limbic and paralimbic dysfunction in psychopathy.

### Conflict of interest statement

The Reviewer, Dr. Decety declares that, despite having collaborated with Dr. Kiehl, the review process was handled objectively. The authors declare that the research was conducted in the absence of any commercial or financial relationships that could be construed as a potential conflict of interest.
