# Supplementary material for: Neural correlates of moral and non-moral emotion in female psychopathy
Source: Front Hum Neurosci. 2014 Sep 25;8:741. doi: 10.3389/fnhum.2014.00741 (PMC4174863; doi:10.3389/fnhum.2014.00741)
Supplement: Supplementary file 1 [file DataSheet1.DOCX]

***Supplementary Material***

**Neural correlates of moral and non-moral emotion in female psychopathy**

**Carla L. Harenski^1^*, Bethany G. Edwards^1^, Keith A. Harenski^1^, and Kent A. Kiehl^1,2^**

^1^ The nonprofit Mind Research Network (MRN) & Lovelace Biomedical and Environmental Research Institute (LBERI), Albuquerque, NM, USA

^2^Departments of Psychology and Neuroscience, University of New Mexico, Albuquerque, NM, USA

*** Correspondence:** Carla L. Harenski, The Mind Research Network, 1101 Yale Blvd NE, Albuquerque, NM, USA

[charenski@mrn.org](mailto:charenski@mrn.org)

1. **Psychiatric disorders across participants**

Below we present details of DSM Axis I and II disorders across all incarcerated participants that completed the study, as well as participants who volunteered but were excluded due to a current Axis I diagnosis. Community control volunteers were excluded from the study if they endorsed the number of items that necessitated administering any full SCID I or II module.

**Table S1.** DSM-IV Axis I and II diagnoses in incarcerated individuals.

|  | **% Study participants with diagnosis^a^** | **# Excluded for current diagnosis** |
| --- | --- | --- |
| **AXIS I** |  |  |
| Mood Disorder |  |  |
| *Bipolar* | 0 | 1 |
| *Major Depression* | 18 | 4 |
| Psychotic Disorder (lifetime) | 0 | 25 |
| Anxiety Disorder |  |  |
| *Panic* | 3 | 2 |
| *Phobia* | 0.5 | 0 |
| *Obsessive-Compulsive* | 1 | 3 |
| *Post-traumatic Stress* | 1 | 4 |
| *Generalized* | 0 | 4 |
| Substance Use Disorder |  |  |
| *Alcohol* | 66 | 0 |
| *Sedative-Hypnotic-Anxiolytic* | 8 | 0 |
| *Cannabis* | 32 | 0 |
| *Cocaine* | 30 | 0 |
| *Amphetamine* | 35 | 0 |
| *Opioid* | 17 | 1 |
| **AXIS II** |  |  |
| Avoidant PD | 1 | --- |
| Dependent PD | 1 | --- |
| Obsessive-Compulsive PD | 0 | --- |
| Paranoid PD | 3 | --- |
| Schizotypal PD | 0 | --- |
| Schizoid PD | 4 | --- |
| Narcissistic PD | 0 | --- |
| Borderline PD | 1 | --- |
| Histrionic PD | 2 | --- |
| Antisocial PD | 40 | --- |

^a^ All Axis I disorders refer to past diagnoses.
